# High focal adhesion kinase expression in breast carcinoma is associated with lymphovascular invasion and triple-negative phenotype

**DOI:** 10.1186/1471-2407-14-769

**Published:** 2014-10-17

**Authors:** Vita M Golubovskaya, Lourdes Ylagan, Austin Miller, Melissa Hughes, Jason Wilson, David Wang, Elizabeth Brese, Wiam Bshara, Stephen Edge, Carl Morrison, William G Cance

**Affiliations:** Department of Surgical Oncology, Roswell Park Cancer Institute, Elm&Carlton Streets, Buffalo, NY 14263 USA; Department of Pathology, Roswell Park Cancer Institute, Buffalo, NY USA; Department of Biostatistics, Roswell Park Cancer Institute, Buffalo, NY USA; Cell Stress Biology, Roswell Park Cancer Institute, Buffalo, NY USA

**Keywords:** FAK, Breast, TMA, Prognosis

## Abstract

**Background:**

Focal adhesion Kinase (FAK) is a nonreceptor protein tyrosine kinase that is overexpressed in tumors and plays a significant role in tumor survival and metastasis. The purpose of the study is to perform correlation of FAK expression with patient prognostic factors using tissue microarrays (TMA) samples.

**Methods:**

We analyzed FAK expression by immunohistochemical staining in 196 breast primary tumor samples from stage II-IV patients and in 117 metastatic tissues matched to the primary tumors using TMA that were stained with FAK monoclonal antibody.

**Results:**

High FAK expression in primary tumors was associated with a younger age of patients (p = 0.033), lymphovascular invasion (p = 0.001) and with the triple-negative phenotype (p = 0.033). FAK expression in 117 metastatic tissues positively correlated with FAK expression in matched primary tumors by Spearman correlation analysis. In addition, a strong positive correlation was observed between high FAK expression and shorter overall survival and progression free survival in patients with metastatic tumors.

**Conclusions:**

The data demonstrate a high potential for FAK as a therapeutic target, especially in triple-negative breast cancer patients with high FAK expression.

## Background

Focal Adhesion Kinase (FAK) is a 125 kDa non receptor kinase which localizes at the focal adhesion sites and is important for tumor survival, metastasis and angiogenesis [1–3]. FAK was shown to be overexpressed in many types of tumors: colon [4], thyroid [5], pancreatic [6], ovarian [7], brain cancer [8], neuroblastoma [9], oral cancer [10], and others. FAK was shown to be associated with an aggressive phenotype in breast cancer tumors [11] and to be overexpressed at early stages of breast tumorigenesis [12], suggesting that FAK expression precedes invasion and metastasis, which is necessary for tumor survival signaling.

In a previous report evaluating FAK levels in 629 breast tumor samples from patients with mostly Stage I or II breast cancer, 25% had high FAK expression, while approximately 75% were classified as having not high FAK levels (11). Those with high FAK expression were noted to have significant associations with poor prognostic features such as higher mitotic index, higher grade, estrogen receptor (ER) and progesterone receptor (PR) negativity, as well as HER-2 overexpression. In the present report, we analyzed FAK expression in 196 stage II-IV breast cancer patients and 117 metastatic samples available matched to the primary tumors using the tissue microarray (TMA) technique which allowed us to analyze hundreds of tissue samples simultaneously using one paraffin block, reducing variability between samples in the case of regular immunohistochemical staining of many samples of large tumor sections [13]. The purpose of this study was to define a method for quantifying FAK levels in TMA breast tissue samples and to identify patient and tumor prognostic factors associated with high FAK expression in more advanced primary and metastatic breast tumor samples.

## Methods

### Tumor samples

Breast cancer tumor samples were collected and all cases of breast cancer diagnosed between November 1994 and January 2008 treated surgically at Roswell Park Cancer Institute (RPCI), Buffalo, NY. Samples were frozen immediately in liquid nitrogen, and other tissue available from blocks fixed in paraffin. Medical records were reviewed to define clinical and pathological characteristics of all cases with tumor samples used for the study. The Roswell Park Cancer Institute Institutional Review Board (IRB) approved this research and approval is consistent with federal, state and local requirements. The clinical and outcome data were de-identified.

### Tissue microarrays (TMA)

TMA’s were constructed from formalin-fixed paraffin tissues with tumors grouped based on hormone receptor and HER-2 status. TMA’s containing breast cancer tumors from 196 breast tumor samples were prepared with each tumor in triplicate. Among these samples, 117 samples included tissue from the primary breast tumor and from matched metastatic tumor tissue. Three one-millimeter tissue cores from formalin-fixed paraffin embedded donor blocks were precisely arrayed into a new recipient paraffin block that included tumor specimens and controls, which included multiple cores of normal tissue from 10 different organs. Each patient had three breast tumor tissue cores on a single TMA slide.

### Immunohistochemical staining

The immunohistochemical staining was performed with FAK 4.47 antibody (Millipore #05-537). For antigen retrieval, slides were heated in the microwave for 10 minutes in citrate buffer (pH 6.0), followed by a 15 minute cool period. Endogenous peroxidase was quenched with aqueous 3% H_2_O_2_ for 10 minutes and washed with 1×PBS with 0.5% Tween 20 solution. Slides were loaded on a DAKO autostainer and blocked with serum-free protein block solution (Dako #X0909) which was applied for 5 minutes and then FAK primary antibody (Millipore #05-537) was applied for one hour. The biotinylated goat anti-mouse IgG (Jackson Immuno Research Labs, #115-065-062) was applied for 30 minutes, followed by the Elite ABC Kit (Vectastain, #PK-6200) for 30 minutes, and the DAB chromagen (Dako, #K4007) for 5 minutes. The slides were counterstained with hematoxylin, dehydrated, cleared and cover slipped.

### Immunohistochemistry scoring for FAK

The scoring was performed by a board-certified pathologist (L.Y.), as described [14]. The scoring system of triplicate tumor cores included intensity of staining (0, none; 1+, weak; 2+, moderate; 3+, strong) plus extent of staining, which was equal to the number of cores with a positive staining (extent 0, no staining in three cores; 1, only one core had a positive staining; 2, only 2 cores had a positive staining; 3, all three cores had the positive staining). Thus, the score ranged from 0 to 6 and included the average intensity and extent of staining.

### Statistical methods

The associations between FAK scores and categorical factors were assessed using independent sample permutation t-test. Patients with less than two tissue sample cores were excluded from analysis. The median FAK score was used to dichotomize patients into FAK high and FAK low categories. A Cutoff value of 4.0 that was equal to median FAK score in 196 primary tumors was used for tumor classification and patient dichotomizing into two groups. The same cut-off for patient dichotomizing into two groups was used for 117 matched primary and metastatic tissues. Fisher’s Exact and the Wilcoxon Rank sum test were used for categorical and continuous comparisons between dichotomous FAK categories. The Spearman correlation analysis was performed in 117 matched primary and metastatic tissues. Normal and Kernel distribution was performed on these samples for FAK expression. Kaplan Meier curves and Logrank test were used for Overall Survival (OS) and progression free survival (PFS) data. The p-value less than 0.05, was considered statistically significant.

## Results

TMA’s contained breast tumor samples from 196 patients with a median age of 56 years, range 27 - 91 years (Table 1). The median number of resected lymph nodes was 19 and median number of positive lymph nodes 4. 68.2% of women had stage II disease and 31.8% had stage III or IV disease (Table 1).

**Table 1:** **Characteristics of breast cancer patients analyzed for FAK staining using TMA**

Characteristic	Number (%)
**Number of cases 196**	
Age (median)	56 (range 27-91)
Positive lymph nodes (median)	4 (range 0-45)
**Histological grade**	
Grade I (well differentiated)	7 (3.7%)
Grade II (moderately differentiated)	36 (19.1%)
Grade III (poorly differentiated)	145 (77.1%)
**Stage**	
Stage IIA	58 (29.7%)
Stage IIB	75 (38.5%)
Stage IIIA	23 (11.8%)
Stage IIIB	26 (13.3%)
Stage IIIC	4 (2.1%)
Stage IV	9 (4.6%)
**Estrogen receptor (ER)**	
Positive	155 (79.9%)
Negative	39 (20.1%)
**Progesterone receptor (PR)**	
Positive	112 (57.7%)
Negative	82 (42.3%)
**HER-2**	
Positive	41 (21.2%)
Negative	152 (78.8%)
**Triple Negative status**	
No	168 (87.5%)
Yes	24 (12.5%)
**Lymphovascular invasion (LVI)**	
No	91 (49.5%)
Yes	93 (50.5%)

**Figure 1:**
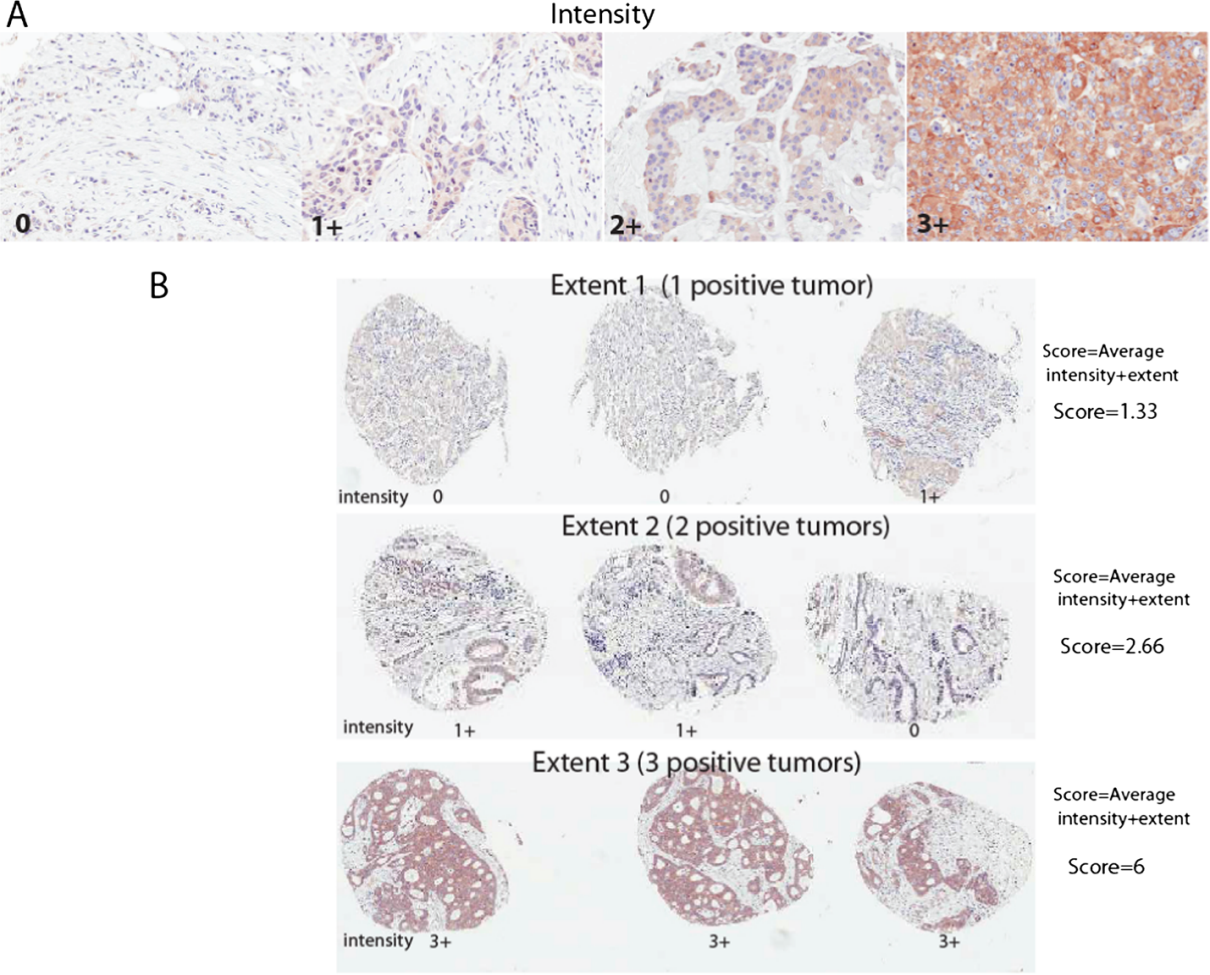
**Immunohistochemical analysis of FAK expression in breast cancer TMA samples.** FAK expression was detected by immunohistochemical staining with FAK 4.47 monoclonal antibody of TMA samples with triplicate tissue cores per tumor stained. The slides were scored by a board-certified pathologist using the following scoring system that measured intensity: (0, none; 1+, weak; 2+, moderate; 3+, strong) **(A)** and extent of staining (0, no positive staining; 1, one core has positive staining score; 2, two cores have positive staining; and three cores have positive staining **(B)**. The staining score was calculated by adding the average intensity and extent of staining and the core ranged from 0 to 6. Upper panel: different intensity of staining from 0 to 3+ is shown in breast samples. The intensity from 0 to 3 is shown on **A** and extent from 1 to 3 is shown on **B** panel.

**Figure 2:**
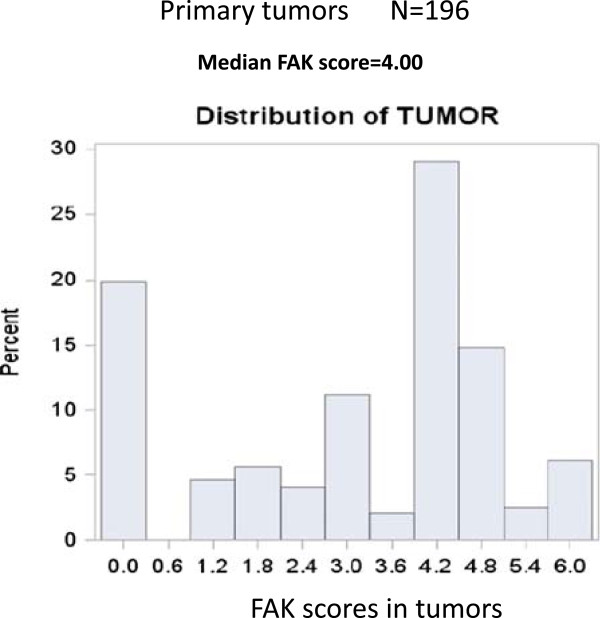
**Distribution of FAK staining in primary tumors.** The medium FAK staining is equal to 4 in primary tumors. The primary tumors were divided for analysis on two groups higher than medium >4 and ≤4 (Table 2).

FAK expression was determined by immunohistochemical staining of the TMA’s tumor tissues, which contained in triplicate three 1 mm cores of each tumor sample. We used the scoring system from 0 to 6, which quantified the average intensity of staining and extent of staining in triplicate TMA samples of each tumor (Materials and Methods). The tumor intensity levels from 0 to 3 are shown in Figure 1A and extent from 1 to 3 on Figure 1B. We dichotomized FAK expression in two groups based on the median FAK staining score in tumors, which was equal to 4 (Figure 2, left panel). One group included tumors with high FAK expression (>4 score) and another group included tumors with low FAK expression (≤4 score) (Table 2). High FAK expression was observed in 27% of patients and low FAK expression was observed in 73% of patients (Table 2).

**Table 2:** **Correlation between FAK expression and clinicopathological characteristics in primary tumors**

Characteristic	FAK ≤ 4 low	FAK > 4 high	Total number	P-value
Number of cases	143 (73%)	53 (27%)	196	
Age (median)	57	51	56	**0.033**
Positive Lymph nodes (median)	3.0	5.0	4.0	0.062
**Histological grade**				
Grade I (well differentiated)	7 (5.2%)		7	0.143
Grade II (moderately differentiated)	23 (17.0%)	13 (24.5%)	36	
Grade III (poorly differentiated)	105 (77.8%)	40 (75.5%)	145	
**Stage**				
Stage IIA	48 (33.8%)	10 (18.9%)	58	0.124
Stage IIB	52 (36.6%)	23 (43.4%)	75	
Stage IIIA	12 (8.5%)	11 (20.8%)	23	
Stage IIIB	20 (14.1%)	6 (11.3%)	26	
Stage IIIC	3 (2.1%)	1 (1.9%)	4	
Stage IV	7 (4.9%)	2 (3.8%)	9	
**Estrogen receptor (ER)**				
Positive	115 (81.6%)	40 (75.5%)	155	0.346
Negative	26 (18.4%)	13 (24.5%)	39	
**Progesterone receptor (PR)**				
Positive	86 (61.0%)	26 (49.1%)	112	0.134
Negative	111 (79.3%)	41 (77.4%)	152	
**HER-2**				
Positive	29 (20.7%)	12 (22.6%)	41	0.770
Negative	111 (79.3%)	41 (77.4%)	152	
**Triple negative status**				
No	126 (90.6%)	42 (79.2%)	168	**0.033**
**Lymphovascular invasion (LVI)**				
No	75 (56.8%)	16 (30.8%)	91	**0.001**
Yes	57 (43.2%)	36 (54.4%)	93	

The statistical significant correlation is marked by bold font.

High FAK expression in primary tumors was associated with younger patient age (the median age was 51 versus 57 with low FAK expression) (p = 0.033) (Table 2). The median number of positive lymph nodes was higher in the group with high FAK: 5.0 versus 3.0 (p = 0.062). There were no statistically significant differences between FAK expression and stage and tumor grade. There were no significant associations between ER negative, PR-negative or Her-2 positive phenotype , but there were significant associations of high FAK expression with triple-negative phenotype (p = 0.033). There was also a highly significant association of high FAK expression with lymphovascular invasion (LVI) (p = 0.001) (Table 2).

**Figure 3:**
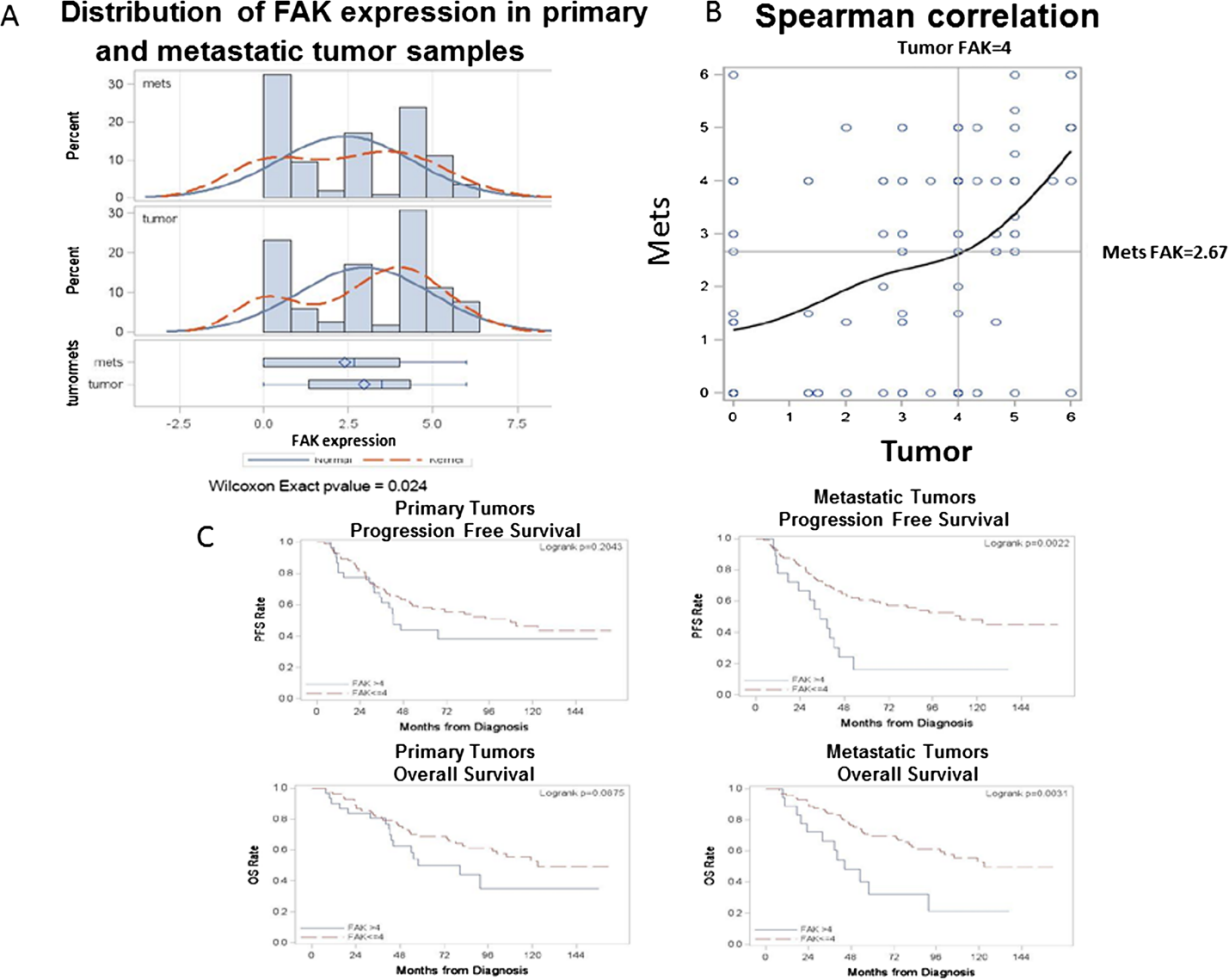
**Analysis of FAK expression in primary tumors and matched metastatic tissues. A**. Distribution of FAK expression in 117 primary and matched metastatic tissues. Upper and lower panels show distribution of FAK expression in primary and metastatic tissues (N = 117). The median FAK expression in primary tumors is equal to 3.5 and in metastatic tissues 2.67 (N = 117). Normal (marked by solid line) and Kernel (marked by dotted line) distribution of FAK expression in both groups are shown. Wilcoxon Exact p-value = 0.024. **B**. Correlation between FAK expression in primary and metastatic tissues. The positive Spearman correlation between FAK expression is shown. Correlation coefficients are shown on left lower panel. **C**. Overall survival and progression free survival in patients with matched primary and metastatic breast cancer tumors. Left panels: Progression-free survival and overall survival (OS) curves in patients with primary tumors with high FAK (FAK > 4) compared to low FAK expression (FAK ≤ 4). Right panels: Progression-free survival curve and overall survival curves in patients with metastatic tumors. There is a strong correlation between high FAK expression in metastatic tumors and shorter PFS and OS. Logrank p < 0.05.

Of the overall 196 patients with primary tumor samples, there were 117 who also had matched metastatic tumors samples. Although these 117 primary tumor samples had higher percentages of being ER and PR negative, HER2 positive, triple negative and having LVI compared to the overall group of 196, this did not reach statistical significance (not shown). We performed analyses of FAK expression in the 117 metastatic tumors that were available for 117 matched primary tumors (Figure 3A). The median score of FAK expression in 117 primary tumors was 3.5 and the median score in matched metastatic tissues was 2.67. Among 117 patients 27 (23%) had FAK score in primary tumors equal to 0, and among these 27 patients with FAK-negative primary tumors 11 (40.7%) had increased FAK expression in metastatic tumors and 16 (59.3%) had score equal to 0 in metastatic tumors. The FAK scores in these metastatic tumors ranged from 1.33 to 6 with mean score equal to 1.2 versus 0 in primary tumors, p < 0.01. The distribution of FAK expression in primary and metastatic tissues is shown in (Figure 3A, upper and lower panels). We dichotomized patients into two groups with the same criteria as used for 196 patients in Table 2, high FAK > 4 and low FAK ≤4 and performed correlation between FAK expression and patient clinical data. Although, there were no statistically significant correlations between FAK expression and patient clinical data in the metastatic samples (not shown), there was a positive Spearman correlation between high FAK expression in primary and metastatic tissues (r = 0.45; p < 0.0001) (Figure 3B). The patients with higher FAK expression in primary tumors expressed higher expression of FAK in metastatic tumors (Figure 3B).Moreover, we performed correlation analysis of FAK expression in matched primary and metastatic tumors with progression free survival (PFS) and overall survival (OS). Figure 3C shows the Kaplan-Meier overall survival and progression free survival curves in primary and metastatic tumors. Although there was a trend towards a worse outcome, there was no statistically significant difference between high FAK expression in primary tumors and PFS and OS. The median overall survival of patients with FAK score >4 in primary tumor was 80 months from diagnosis, while those with FAK score ≤4 was 123.0 months (logrank p-value = 0.0875). The same was observed with progression free survival that was equal to 42.6 months in patients with high FAK expression versus 107.9 months with low FAK expression (logrank p = 0.2) (Figure 3C, left panels). In contrast, there was a strong statistically significant difference between high FAK expression and PFS and OS in patients with metastatic samples (Figure 3C, right panels). The progression free survival in patients with high FAK expression (FAK > 4) in metastatic tissues was 35 months, while PFS in patients with low FAK expression in metastatic tumors was 110.9 months (logrank p = 0.002). The overall survival in patient with high FAK expression in metastatic tumors was 44 months versus 123 months in patients with low FAK expression, (logrank p = 0.003). Thus, although there was no correlation between FAK expression in primary tumors, there was a strong positive correlation between high FAK expression and shorter PFS and OS in patients with metastatic tumors that may be very important for clinical studies.

## Discussion

Overexpression of FAK has an important role in tumorigenesis, tumor survival, and metastasis. This paper highlights a method of accurately measuring FAK expression using TMA technique, allowing direct comparison of FAK expression in multiple tissue samples. The prognostic value of FAK and its associated clinicopathologic characteristics is important for development of new and more effective treatment regimens for breast cancer patients, especially as FAK inhibitors enter clinical trials.

A recent report that analyzed FAK expression in 98 breast tumor samples using TMA analysis did not show a significant association with FAK and prognostic indicators in breast cancer [15]. That study used a different FAK antibody (rabbit polyclonal against phospho Y397 FAK) [13] in contrast to the FAK 4.47 monoclonal antibody used in this study, which was shown before to be highly specific to FAK [4]. In addition, the present study examined almost two times more breast cancer samples, used each tumor in triplicate, and the scoring system of triplicate samples included not only the average intensity but also the extent of staining, allowing for more accurate analysis. The distribution of high FAK expression being observed in 27% of patients and low FAK expression in 73% of patients in this paper is consistent with the findings of previous reports and validates this method of analyzing FAK expression (11).

Our previous report [11] demonstrated an association between high FAK expression and overexpression of Her-2, while this report did not. This difference may have a few possible explanations. First, between the two reports there are differences in the stage of tumors that were analyzed. While the present report did not include patients with stage I disease and instead analyzed patients with stage II (68.2%) and stages III and IV (31.8%) breast cancer, the previous report analyzed patients at earlier stages and included 39% of patients with stage I, 50% with stage II, and only 11% patients at stage III and IV disease. Therefore, correlation of high FAK and Her-2 was observed at earlier stage of disease, but not at later stage of disease. Second, the smaller sample size in this report compared to the prior report (196 vs 629 patients) may have impacted the results of the Her-2 association. Lastly, the way in which FAK levels were scored in this paper differs from that of previous reports potentially contributing to these differences.

Although a significant association was not found between FAK and Her-2 in this paper, there is evidence that FAK and Her-2 signaling pathways are linked in breast cancer development. Activated FAK colocalized with Erb-2/3 receptors at cell protrusions and FAK signaling had an essential function in ErbB-induced invasiveness, metastasis and oncogenesis [16]. A recent report addressed the issue of anti-Her-2 agent resistance in breast cancers where targeting of FAK with FAK inhibitor PF4554878 and Her-2 with trastuzumab in ER+/Her-2+ patients showed synergistic effect on the suppression of cell growth and improved the response to trastuzumab [17].

This report for the first time demonstrated high levels of FAK in triple-negative breast cancer patients and correlated with shorter overall survival of the triple-negative group (not shown). This association can be important for the development of therapies for triple-negative breast cancer, suggesting an important role of FAK in triple-negative breast cancer survival signaling. This data is consistent with previous data on increased FAK gene amplification by FISH analysis in triple-negative breast cancer tissues [18]. Since triple-negative breast cancer tumors tend to be more aggressive and result in a worse prognosis, more effective therapies are needed in this population of patients [19]. Emerging data show that FAK can be an effective therapeutic target in tumors, especially in highly aggressive triple-negative breast cancer tumors. In fact, we tested MDA-231 triple-negative breast cancer cells with FAK autophosphorylation inhibitor [6, 20, 21] and showed that this FAK inhibitor significantly decreased cancer cell viability and clonogenicity *in vitro* (not shown). Thus, these data suggest that targeting FAK in triple-negative breast cancer patients is a promising approach. It is important to note that FAK has many binding partners and integrates multiple oncogenic survival pathways and sequesters tumor-suppressor pathways [1, 22]. Therefore, future therapeutics should involve multiple targets cross-linked with FAK survival signaling in breast cancer tumors, and especially in aggressive triple-negative breast cancer tumors.

The data on association of high FAK expression in primary breast cancer tumors with lymphovascular invasion supports the important role of FAK in epithelial and mesenchymal transition [23], angiogenesis, lymphangiogenesis [24] and metastasis [25]. We detected correlation between high FAK expression and lymphovascular invasion, which is consistent with the role of FAK in metastasis and angiogenesis/lymphangiogenesis. The association of high FAK expression and lymphovascular invasion in tumors correlated with worse patient prognosis and lower overall survival. Metastasis of breast cancer occurs mainly through lymphatic system and the dissemination of tumor cells to the regional lymph nodes is an indicator of breast cancer aggressiveness [26]. The recent study found that LVI was significantly associated with predicting patient outcome leading to shorter breast cancer specific survival as well as distant metastasis-free survival [27].Interestingly, we detected high level of FAK in metastatic samples with the median score FAK staining equal to 2.67, which was lower than in primary tumors (median score in 117 primary tumors was 3.5 (Figure 3). While we did not find correlation between clinicopathological data such as hormone receptor status, triple-negative phenotype or LVI in metastatic tissues that were identified in the matched primary tumor samples, we did find correlation between high FAK expression in primary tumors and metastatic tissues. In addition, among 117 patients 23% had FAK-negative primary tumors, and among these more than 40% increased FAK in metastatic tumors confirming important role of FAK in metastasis. Although the median level of FAK was not increased in metastatic tissues compared to the matched primary tumors, we found a strong positive correlation between high FAK expression in metastatic samples and shorter progression-free survival and overall survival that was not observed in the primary tumors. The overall survival of patients with high FAK expression was almost 3-fold shorter (44 months versus 123 months from diagnosis) than in patients with low FAK expression. These data support the important role of FAK in metastasis.

## Conclusions

The present simultaneous analysis of FAK expression using tissue microarrays allows for a more comprehensive method of analyzing FAK expression in breast tumor samples. This analysis demonstrated the prognostic value of high FAK expression in breast tumors being associated with more aggressive tumor features such as lymphovascular invasion and triple-negative phenotype. In addition, a high positive correlation between high FAK expression in primary tumors and metastatic tissues was shown, with significantly worse overall and progression free survivals found in patients whose metastatic tumors had high FAK expression. These associations are important for understanding the mechanisms of breast tumorigenesis and for the development of novel biomarkers associated with FAK overexpession and new, effective anticancer therapies.

## Abbreviations

FAK: Focal Adhesion Kinase
LVI: Lymphovascular invasion
OS: Overall survival
PFS: Progression free survival
TMA: Tissue microarray.
